# A neurophysiological perspective on the integration between incidental learning and cognitive control

**DOI:** 10.1038/s42003-023-04692-7

**Published:** 2023-03-27

**Authors:** Adam Takacs, Christian Beste

**Affiliations:** 1 Cognitive Neurophysiology, Department of Child and Adolescent Psychiatry, Faculty of Medicine, Technical University of Dresden, Dresden, Germany; 2 University Neuropsychology Center, Faculty of Medicine, Technical University of Dresden, Dresden, Germany

**Keywords:** Cognitive control, Human behaviour

## Abstract

Adaptive behaviour requires interaction between neurocognitive systems. Yet, the possibility of concurrent cognitive control and incidental sequence learning remains contentious. We designed an experimental procedure of cognitive conflict monitoring that follows a pre-defined sequence unknown to participants, in which either statistical or rule-based regularities were manipulated. We show that participants learnt the statistical differences in the sequence when stimulus conflict was high. Neurophysiological (EEG) analyses confirmed but also specified the behavioural results: the nature of conflict, the type of sequence learning, and the stage of information processing jointly determine whether cognitive conflict and sequence learning support or compete with each other. Especially statistical learning has the potential to modulate conflict monitoring. Cognitive conflict and incidental sequence learning can engage in cooperative fashion when behavioural adaptation is challenging. Three replication and follow-up experiments provide insights into the generalizability of these results and suggest that the interaction of learning and cognitive control is dependent on the multifactorial aspects of adapting to a dynamic environment. The study indicates that connecting the fields of cognitive control and incidental learning is advantageous to achieve a synergistic view of adaptive behaviour.

## Abstract (web summary)

EEG analysis in participants carrying out a cognitive conflict task with embedded sequential regularities indicates that the integration of incidental learning and cognitive control aids our understanding of related adaptive behaviour

## Introduction

Changing the course of action to meet our goal requires effort, therefore, it is paramount to recognise when it is necessary to do so^1–4^. A collection of functions, often labelled as cognitive control, can be recruited to meet one’s goal^5,6^. For instance, when stimulus properties trigger incompatible stimulus-response (S-R) representations or response tendencies, detection of the cognitive conflict typically evokes higher-order cortical processes to redirect behaviour in a goal-directed manner^1,2,7^. Cognitive control’s main function is to adjust the engagement of effortful, task-related mechanisms in accordance with the mentally held task set^2^. Monitoring cognitive conflict is not an isolated process, as it is also influenced by contextual cues^3,7–12^. Through repetition, regularities in the context can be learnt and subsequently used to build predictions on the upcoming demand for cognitive control^9,10,13^. Here we investigated how incidental learning influences cognitive control at the levels of behavioural adaptation and neurophysiological signatures. The current study aimed to connect current trends in the fields of cognitive control and incidental learning, and by doing so we promote an integrated view between the implications of intentional and incidental forms of adaptive behaviour^14^.

Imagine that you learn a new dance with a partner. As you gain more expertise, your moves follow a predefined sequence of steps and executing them will not require much attention. At the same time, you also need to anticipate when your partner would like to change directions and adapt your steps accordingly. If you are preoccupied with how and when to make changes, the dance will never be smooth. If you do not notice the changes at the right time, one of yours might leave the dance floor with an aching foot.

In a lab environment without a dance floor, a typical paradigm to study cognitive control is the colour-word Stroop task^15^. Here, participants are asked to respond to the colour of the stimulus while ignoring the meaning of the word. The congruency between the perceptual and semantic information (i.e., the word “blue” written in blue colour) facilitates response selection compared to a neutral condition (i.e., blue colour target without a semantic association). However, incongruency between the task-relevant colour and task-irrelevant semantic stimulus dimensions (i.e., the word blue written in yellow colour) leads to more errors and slower responses.

Contextual cues can modulate performance in a Stroop task. For instance, the list-wide proportion congruence (LWPC) effect^7,8^ is observed through the manipulation of the overall ratio between congruent and incongruent trials. If the trials are predominantly incongruent (high level of conflict), conflict-related response cost is reduced in comparison to a predominantly congruent set of trials (low level of conflict)^7,8^. That is, distributional statistics of the stimuli influence cognitive control. An example of trial-by-trial contextual adaptation is the congruency sequence effect (CSE). Smaller conflict-related response cost is observed after a high conflict trial (incongruent) than after a low conflict event (congruent)^7,10^. That is, recent memory on the conflict level has predictive power on subsequent conflict detection and response control^10^. The robustness of CSE raises the question of whether transitions between Stroop trials can be learnt and if yes, does learning of conditional probabilities contribute to cognitive control? In a modified Stroop task, when sequential probabilities predicted the next stimulus’ colour, a smaller congruency effect was detected compared to a random series of trials^16^. Thus, incidental acquisition of transitional probabilities and cognitive control may engage in a cooperative fashion. Consequently, both distributional and conditional probability information could influence cognitive control by building predictions on conflict demand to reduce the related response cost.

This cooperative mode has been put forward by the notion of control state associations^10^. It has been proposed^10^ that cooperation between cognitive conflict and incidental learning processes occurs through abstract categories or “control states”. Control states (C) represent the allocation of top-down resources, such as visual attention or response inhibition^17^. A control state can integrate (i) internal models of the task goal, (ii) implicit memories associated with the current S/R/C features, and (iii) pre-existing biases (i.e., habits, stereotypical responses, etc.)^17^. Control states are represented in an associative S-C network similar to the network of S-R associations^9,10,18^. Through these networks, a specific stimulus can activate a context-appropriate control state^13^. Whenever predictions based on S-R properties are sufficient to guide the behaviour, the control state representation does not need to be involved. However, if the original (lower level) predictions fail, the association between the appropriate control state and stimulus contingency has to be used^8–10,13^. These bindings between contextual information (stimulus) and control state are the so-called S-C event files or “episode files”^10^. Incidental learning of S-C contingencies can potentially predict the need for top-down engagement and alleviate response adaptation. Conflict detection can also serve as a teaching signal to promote learning by modifying the focus of attention or prioritising the sensory input^4,9,19^. In sum, S-C representations allow cooperation between incidental learning and intentional control processes.

**Fig. 1:**
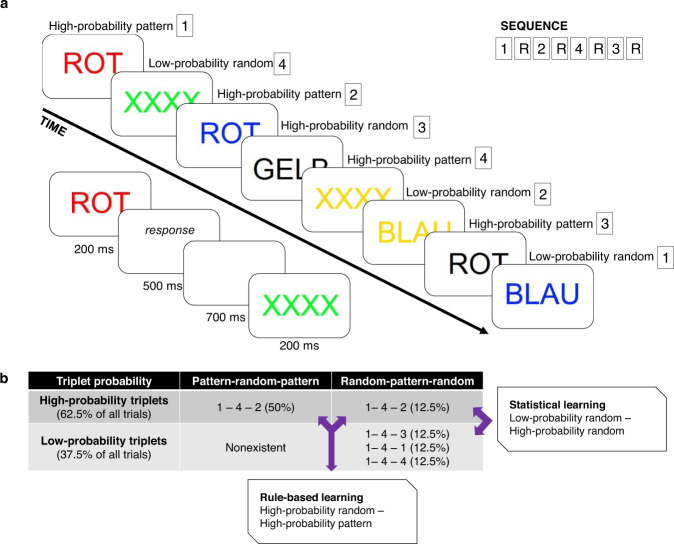
Schematic illustration of the alternating sequential Stroop task in Experiments 1 and 2. Participants saw a word or coloured “XXXX” characters in the middle of the screen. **a** The stimulus presentation followed an eight-element sequence, in which pattern and random (R) elements alternate. Numbers denote the four possible conflict conditions (1—congruent, 2—incongruent, 3—word naming, 4—colour naming). The timing of the task is presented on the left side of the figure. **b** Some series of consecutive elements (triplets) were more probable in the task than others. High-probability triplets could either end with a pattern or with random elements, while low-probability triplets always end with a random element. Two types of sequence learning performance can be calculated in the task. Statistical learning is the difference between high- and low-probability random elements. Rule-based learning is the difference between pattern elements (presented according to the serial order) and random elements (not determined by the sequence).

In a recent study^20^, Stroop trials were presented according to either colour-based (S-R) or congruency-based (S-C) sequential regularities. Interestingly, the two groups showed comparable effects of conflict. However, predictability facilitated responses only in the S-R group, and this effect was independent of congruency. Thus, the study^20^ did not support cooperation between learning and control through S-C predictability. However, it is possible that the brief training did not allow the participants to form statistical memories that are more complex than the distribution of congruent and incongruent conditions. Therefore, the current study followed training protocols in which participants were able to learn transitional relations incidentally^21,22^. Another consideration was that conditional probabilities can define relations not only between adjacent sequential items (first-order transitions) but also between non-adjacent ones (second-order transitions)^23–26^. The two forms of learning also involve different neurocognitive mechanisms: since the processing of non-adjacent relations necessitates the suppression of the interleaving item, it has been thought that learning of non-adjacent dependencies requires an engagement of top-down processes^24^. In contrast, learning of the more simple adjacent dependencies is a purely automatic, bottom-up mechanism^24,27,28^. In the current study, S-C relations were defined as non-adjacent, second-order transitions in contrast to the adjacent dependencies used by Jimenez et al.^20^. Figure 1 provides details about the experimental approach.

### Multiple coding of regularities: statistical and rule-based learning

Sequence learning is not a unitary construct, but involves different processes that detect, encode and retrieve sequentially presented regularities^24,27,29–31^. A prominent distinction is between the acquisition of probabilistic information (statistical learning) and sequential order^22,26,27,30^. This latter process is called higher-order sequence learning^21,22^, order-based learning^32^ or rule-based learning^26,30^. Within sequence learning, statistical learning can be operationalized as the differentiation between high- and low-probability elements that otherwise carry indistinguishable information about the serial order^22^. In contrast, rule-based learning is the differentiation between elements that are defined by the serial order and those that are presented randomly, while the probability of occurrence remains the same^22,26^. While statistical learning enables the acquisition of distributions and probabilistic interdependence in the stimulus stream, rule-based learning controls and integrates learning results into higher-order regulations^27^.

These learning functions operate in overlapping time windows, however, they have different time courses^32,33^: fast estimation of statistics reaches its plateau early in contrast to the more gradual curve of rule-based learning. In other words, a larger rule-based learning effect can be expected at the end of the task than at the beginning. In contrast, statistical learning shows less variability over time. Moreover, the two learning processes have different interdependence on other cognitive functions^27,30,34^, they are related to different neural sources^26^, and are thought to generate differentiable expectations about upcoming events^30^. Statistical learning is considered to be a more bottom-up process that develops fast in the presence of recurring patterns in the sensory experience^26,27,35^. In contrast, rule-based learning operates with the gradual accumulation of sequential rules, and the learnt associations are also available for top-down processes^22,26,27^. Considering the simultaneous nature of statistical and rule-based learning, the current study will investigate the potential contribution of both processes to cognitive control. The two forms of sequence learning may have different capacities to interact with cognitive control. Due to their simultaneity, it is often hard to disentangle the two processes at the behavioural level, however, the analysis of the concurrent neurophysiological signal can provide confirmation of the behavioural effects and further insights into the mechanisms behind them^26,32,33,36–38^. The current study investigated concurrent incidental sequence learning and cognitive control by considering neurophysiological information that are differentially related to cognitive control and contextual learning.

### Cascade of processes in the neurophysiological signal

In a Stroop task, a frontocentral negative deflection is typically observed with a peak of approximately 450 ms after stimulus presentation^39^. This so-called N450 event-related potential (ERP) component’s amplitude is larger in incongruent than in congruent trials, therefore, it is an ideal candidate to study conflict detection^39–41^. Therefore, the N450 was selected as a more specific marker of conflict detection in the current study. Another benefit of using the ERP approach is the potential to differentiate between a cascade of processes^39,42^. For instance, conflict detection and the retrieval of previously learnt stimulus-response (S-R) associations can be separated by ERPs. Namely, the P3 component, a positive deflection that typically occurs on parietal channels 300–600 ms after stimulus presentation^43^ is sensitive to stimulus probability, habituation, and the time since the last target presentation, which are all implicated in contextual (sequence) learning^44^. In incidental sequence learning, the P3 amplitude is larger for less predictable than for more predictable targets^38^. Moreover, it reflects the retrieval of S-R associations both in incidental learning^38,45–47^ and cognitive conflict tasks^48–50^. Specifically, retrieving a less accessible S-R link would attenuate the P3 amplitude, which then reflects the difficulty of response selection^51^ and the amount of processing resources needed^42,43^. The analyses of both N450 and P3 are useful to distinguish between the processing stages of conflict detection (N450) and decision-making/response selection (P3) and how incidental sequence learning modulates them, respectively. Since both increased conflict and increased task difficulty prolong responses, the two stages would not be differentiable by using behavioural measures alone.

### Hypotheses

In the current study, RT, accuracy, and mean amplitude of the N450 and P3 components will be analysed separately for statistical learning and rule-based learning. If sequential S-C links develop independently from the level of conflict^20^, learning effects would be observed on the P3 but not on the N450, similar to S-R-based sequences^38,45–47^. However, if S-C sequence learning modulates behaviour only in case of high conflict^9,10,16,52^, learning effects would be expected on the N450 but not on the P3. We have expected that learning and integrating abstract information into S-C representations is more effortful than similar processes based on S-R associations^9,10^. Therefore, switching from local binding of S-R features to global binding of S-C episodes is only expected to happen if efficiency in predicting the next event necessitates it^8,10^.

Considering the nature of simultaneous learning functions and their potential predictive value in cognitive control, we had the following expectations. If task complexity leads to frequent errors, participants can learn both statistical and rule-based regularities of S-C episodes^10^, that is, response times will be shorter when statistical or rule-based regularities have high predictability on the trial’s congruency compared to low predictability trials. As statistical learning develops faster than rule-based learning, we expected the statistical learning effects at the behavioural level already in the beginning (first half) of the task. We expected that P3 amplitude in high predictability conditions will be smaller than in low predictability (i.e., statistical learning effect and rule-based learning effect). We expected that N450 amplitude in high predictability conditions will be larger (more negative) than in low predictability conditions if the cognitive conflict necessitates it (i.e., in incongruent condition).

After the analysis of Experiment 1, follow-up behavioural studies were conducted to confirm the replicability and generalisability of the findings of Experiment 1 (see Supplementary Results). Specifically, Experiment 2 was performed as an internal replication effort of Experiment 1. We have expected that learning-related benefit on cognitive control in Experiment 1 can also be observed in an independent group in Experiment 2. Additionally, we have performed Experiment 3 to investigate the generalisability of the findings of Experiments 1 and 2 to other control state constellations. Namely, the task was modified to eliminate the potential effect of switching between dominant response dimensions. Experiment 3 is presented as an exploratory analysis. Finally, Experiment 4 was performed to investigate the generalisability of the findings of Experiments 1 and 2 to another sequence.

## Results

We analysed the interaction between learning of predictabilities and experimental conditions. First, the full factorial design of the experiment was analysed with the within-subject factors of predictability (as triplet types: high-probability pattern, high-probability random, low-probability random), condition (congruent, incongruent, word naming, colour naming), and period (first half and second half). The main effects and interactions are summarised in Tables 1–3. For the sake of brevity, only significant results are described in detail in the Results section. In case of a significant interaction that involved both predictability and condition, follow-up analyses were conducted to quantify how predictability could affect RT, accuracy, or mean amplitude of the N450 and P3 components in different types of conflict situations. This was done according to previous studies with the ASRT paradigm^22,26,32,53,54^, to limit the number of pair-wise comparisons. Specifically, we analysed whether the difference between high-probability random and low-probability random trials (*statistical learning*) and the difference between the high-probability pattern and high-probability random trials (*rule-based learning*) was dependent on the condition. All post hoc comparisons are Bonferroni-corrected.

**Table 1:** Results of three-way repeated-measures ANOVAs with predictability, condition, and period as within-subject factors for RT and accuracy data.

	Factor	*F*	*ε*	*p*	*η*_p_^2^
Behaviour RT	Predictability	0.30	0.787	0.688	0.010
	Condition	114.90	0.811	**<0.001**	0.793
	Period	59.60	–	**<0.001**	0.665
	Predictability × Period	0.83	–	0.440	0.027
	Condition × Period	0.77	–	0.515	0.025
	Predictability × Condition	4.68	–	**<0.001**	0.135
	Predictability × Condition × Period	3.80	–	**0.001**	0.112
Behaviour accuracy	Predictability	3.38	0.728	0.057	0.101
	Condition	48.51	0.591	**<0.001**	0.618
	Period	24.66	–	**<0.001**	0.451
	Predictability × Period	2.31	0.705	0.127	0.071
	Condition × Period	7.85	–	**<0.001**	0.207
	Predictability × Condition	4.44	–	**0.003**	0.103
	Predictability × Condition × Period	0.60	–	0.734	0.019

Significant main effects and interactions are boldfaced and detailed in the main text.

**Table 2:** Results of the condition by period follow-up ANOVAs for statistical learning and rule-based learning for the RT and accuracy data.

		Factor	*F*	*p*	*η*_p_^2^
Statistical learning	Behaviour RT	Condition	3.09	**0.031**	0.093
		Period	1.38	0.250	0.044
		Condition × Period	5.46	**0.002**	0.154
	Behaviour accuracy	Condition	4.53	**0.005**	0.131
		Period	3.06	0.090	0.093
		Condition × Period	1.03	0.382	0.033
	N450 mean amplitude	Condition	2.67	0.052	0.082
Rule-based learning	Behaviour RT	Condition	1.71	0.170	0.054
		Period	0.59	0.449	0.019
		Condition × Period	2.07	0.110	0.064
	Behaviour accuracy	Condition	2.11	0.105	0.066
		Period	0.93	0.343	0.030
		Condition × Period	1.03	0.382	0.033
	N450 mean amplitude	Condition	2.93	**0.038**	0.089

Below the behavioural results, follow-up ANOVAs are reported for statistical learning and rule-based learning for the N450 main amplitude data (without the factor period). Significant main effects and interactions are boldfaced and detailed in the main text.

**Table 3:** Results of three-way repeated-measures ANOVAs with predictability and condition as within-subject factors for P3 main amplitude and N450 main amplitude and onset latency data.

	Factor	*F*	*ε*	*p*	*η*_p_^2^
P3 mean amplitude	Predictability	0.15	–	0.857	0.005
	Condition	10.15	0.884	**<0.001**	0.253
	Predictability × Condition	0.66	0.775	0.646	0.021
N450 mean amplitude	Predictability	1.11	0.705	0.319	0.036
	Condition	2.65	0.764	0.070	0.081
	Predictability × Condition	2.47	0.876	**0.032**	0.076
N450 onset latency	Predictability	1.33	0.884	0.272	0.042
	Condition	0.60	0.776	0.577	0.020
	Predictability × Condition	2.00	0.475	0.123	0.062

Significant main effects and interactions are boldfaced and detailed in the main text.

In these follow-up ANOVAs, we analysed whether the difference between high-probability random and low-probability random trials (statistical learning) and the difference between the high-probability pattern and high-probability random trials (rule-based learning) was dependent on the condition. Please, note, that this step has the same purpose as conducting post-hoc tests on the omnibus (full factorial) ANOVA. The follow-up ANOVAs have the advantage to provide results that can be directly linked to the hypotheses (e.g., statistical learning in incongruent vs statistical learning in colour naming condition) and omit contrasts that are not planned or meaningful in the design (e.g., all possible differences between the high-probability pattern and low-probability random trials). In the text, average and standard error are provided as descriptive values.

### Accuracy data inform about task difficulty

Please note, that RTs and ERPs were quantified by using correctly responded trials only, therefore a direct relationship between accuracy and ERP effects were not central to the hypotheses^33,38^. Nevertheless, we report the accuracy results for the sake of completion and to describe the varying level of difficulty that participants faced in the different conditions. Accuracy rates (percentage of correctly responded trials) were analysed in a three-way repeated-measures ANOVA with predictability, condition, and period as within-subject factors. The main effects and interactions are summarised in Table 1. The main effects of condition (*F*(3, 90) = 48.51, *ε* = 0.591, *p* < 0.001, *η*_p_^2^ = 0.618) and period (*F*(1, 30) = 24.66, *p* < 0.001, *η*_p_^2^ = 0.451) were significant. The accuracy rate was lower in incongruent (70% ± 0.1) than in colour naming (81% ± 0.1, *p* < 0.001) or in congruent trials (85% ± 0.1, *p* < 0.001). Additionally, participants were less accurate in word naming (70% ± 0.1) than in colour naming (*p* < 0.001) or congruent trials (*p* < 0.001). The accuracy rate was lower in colour naming than in congruent trials (*p* < 0.001). The accuracy did not differ significantly between incongruent and word naming conditions (*p* = 0.999). Participants became more accurate for the second period (82% ± 0.1) compared to the first one (72% ± 0.1, *p* < 0.001). The interaction of condition by period was significant (*F*(3, 90) = 7.85, *p* < 0.001, *η*_p_^2^ = 0.207). In the first period, participants had a lower accuracy rate in incongruent (65% ± 0.1) than in colour naming (76% ± 0.1, *p* < 0.001) or in congruent trials (82% ± 0.1, *p* < 0.001). Additionally, participants were less accurate in word naming (64% ± 0.1) than in colour naming (*p* < 0.001) or congruent trials (*p* < 0.001). Accuracy was lower in colour naming than in congruent trials (*p* < 0.001). The accuracy did not differ significantly between incongruent and word naming conditions (*p* = 0.999). Similarly, in the second period, participants made more errors in incongruent (75% ± 0.1) than in colour naming (85% ± 0.1, *p* < 0.001) or in congruent trials (89% ± 0.1, *p* < 0.001). Additionally, participants were less accurate in word naming (78% ± 0.1) than in colour naming (*p* < 0.001) or congruent trials (*p* < 0.001). Accuracy was lower in colour naming than in congruent trials (*p* < 0.001). The accuracy did not differ significantly between incongruent and word naming conditions (*p* = 0.548). The predictability by condition interaction was significant (*F*(6, 180) = 4.44, *p* = 0.008, *η*_p_^2^ = 0.103). In the word naming condition, participants were less accurate in high-probability random trials (68% ± 0.1) than in high-probability pattern trials (71% ± 0.1, *p* = 0.028) and in low-probability random trials (73% ± 0.1, *p* = 0.005). High-probability pattern trials of word naming did not differ significantly from low-probability random word-naming trials (*p* = 0.054). Other pair-wise differences in all other conflict conditions did not differ significantly from each other (*p*s > 0.164). In the case of *statistical learning* (Table 2), participants showed more learning in word naming (−0.05% ± 0.2) than in colour naming (0.001% ± 0.01, *p* = 0.035) and more learning in word naming than in incongruent trials (0.16% ± 0.01, *p* = 0.016). None of the other pair-wise comparisons was significant (*p*s > 0.565). In *rule-based learning*, there were no significant pair-wise differences.

### RT data reveal an interaction between stimulus conflict and sequential regularities

**Fig. 2:**
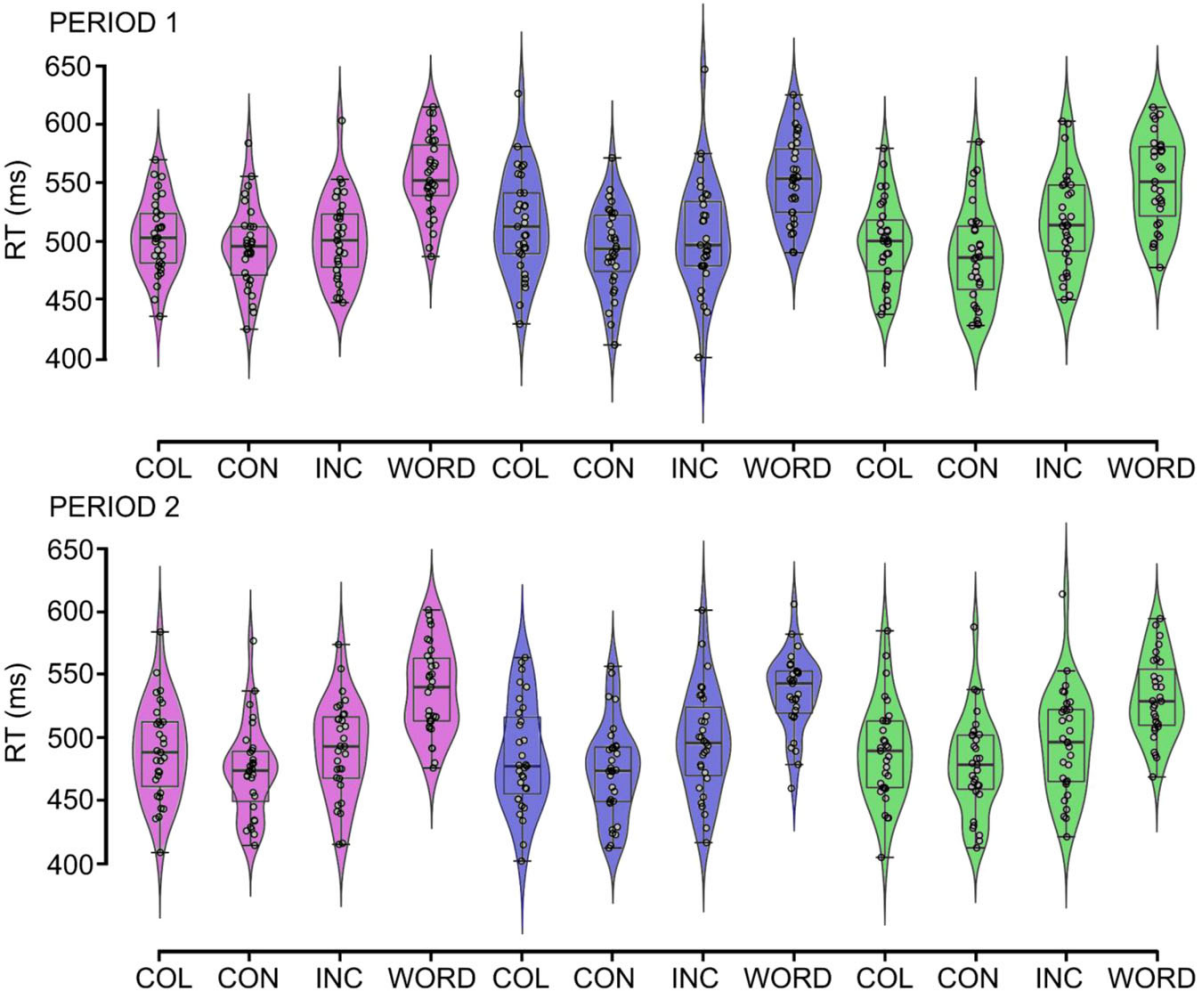
Behavioural results of the main experiment. Individual and group reaction times as a function of predictability, condition, and period (Period 1 and Period 2). Group level RTs are presented as box plots, where the central vertical bar denotes the median. The bottom and top edges denote the 25th and 75th percentile. The kernel probability density of the data is presented as violin elements. Individual RTs are presented as scatterplots. Period 1 and 2 are shown separately. Within each period, RTs are organised as triplet types: high-probability pattern (purple), high-probability random (blue), and low-probability random (green). Stroop conditions are marked on the *X* axis: COL: colour naming, CON: congruent, INCON: incongruent, WORD: word naming. The presented result is based on *N* = 31 participants’ data.

The RT data are shown in Fig. 2.

First, RTs were analysed in a three-way repeated-measures ANOVA with predictability, condition, and period as within-subject factors. The main effects and interactions are summarised in Table 1. The main effects of condition (*F*(3, 90) = 114.90, *ε* = 0.811, *p* < 0.001, *η*_p_^2^ = 0.793) and period (*F*(1, 30) = 59.60, *p* < 0.001, *η*_p_^2^ = 0.665) were significant. Participants responded slower in word naming (520.7 ms ± 5.6) than in incongruent (501.9 ms ± 6.8, *p* < 0.001), congruent, (483.8 ms ± 6.3, *p* < 0.001), or colour naming trials (496.9 ms ± 6.5, *p* < 0.001). Reaction times were longer in incongruent than in congruent trials (*p* < 0.001) which suggests a general congruency (Stroop) effect. Additionally, participants responded slower in colour naming than in congruent trials (*p* < 0.001). Colour naming and incongruent trials did not differ significantly from each other (*p* = 0.798). Participants became faster for the second period (498.2 ms ± 6.2) compared to the first one (515.2 ms ± 5.9, *p* < 0.001). Importantly, both the predictability by condition interaction (*F*(6, 180) = 4.68, *p* < 0.001, *η*_p_^2^ = 0.135), and the three-way interaction between predictability, condition and period were significant (*F*(6, 180) = 3.80, *p* = 0.001, *η*_p_^2^ = 0.112). The interaction effects were further analysed below.

### Evidence for enhanced statistical learning but not rule-based learning under high cognitive conflict

In the case of *statistical learning* (Table 2), the difference between high-probability random colour naming and low-probability random colour naming trials decreased from the first period (15.9 ms ± 3.4) to the second one (−3.7 ms ± 3.9, *p* < 0.001). In the first period, high-probability random colour naming trials (514.9 ms ± 7.8) were slower than low-probability random trials (499.0 ms ± 6.5, *p* < 0.001), which is considered an inverse statistical learning effect. None of the other conditions showed significant changes between the periods (*p*s > 0.070). Importantly, statistical learning was larger in incongruent (−12.7 ms ± 6.1) than in colour naming condition (15.9 ms ± 3.4, *p* = 0.002) in the first period. There were no other significant pair-wise differences between conditions in the two periods (*p*s > 0.165). Thus, statistical learning was larger in incongruent (high conflict) than in colour naming (neutral) trials at the beginning of the task, however, this difference was not significant in the second period. In the case of *rule-based learning*, there were no significant pairwise comparisons (*p*s > 0.074). Thus, the analyses showed enhanced statistical learning when the demand for cognitive conflict was high (i.e., in the incongruent condition).

In sum, RT analyses showed that conflict levels affected participants’ behaviour: compared to the neutral condition, the overlap between stimulus dimensions facilitated responses (congruency effect), while a switch between the dominant response dimension (from perceptual to semantic) led to slower responses. Neither statistical nor rule-based regularities showed a significant effect on RTs, however, the presented regularities interacted with the Stroop conditions. At the beginning of the task, statistical learning was larger in the incongruent condition. In the second half of the task, statistical learning was not modulated significantly by conditions (see Table 3).

Additionally, follow-up experiments were conducted to test the replicability and generalisability of the behavioural results. The results of Experiments 2–4 can be found in the Supplementary Materials (Supplementary Results and Tables 1–3).

### Neurophysiological data: N450 amplitude was modulated by the interaction between stimulus conflict and sequential regularities

Mean amplitude on channel P1 (see Supplementary Fig. 1) in the time window of 280–380 ms was analysed in a two-way repeated-measures ANOVA with predictability and condition as within-subject factors. The main effects and interactions are summarised in Table 3. The main effect of condition was significant (*F*(3, 90) = 10.15, *ε* = 0.884, *p* < 0.001, *η*_p_^2^ = 0.253). The P3 amplitude was smaller in word naming (7.00 µV/m^2^ ± 2.03) than in congruent (10.60 µV/m^2^ ± 2.30, *p* < 0.001) and incongruent (9.40 µV/m^2^ ± 2.22, *p* = 0.019) trials. The other pair-wise differences were not significant (*p*s > 0.073). That is, the P3 amplitude was lower in the condition in which a semantic decision was required (word naming) as opposed to the other two conditions with a perceptual (colour) decision.

**Fig. 3:**
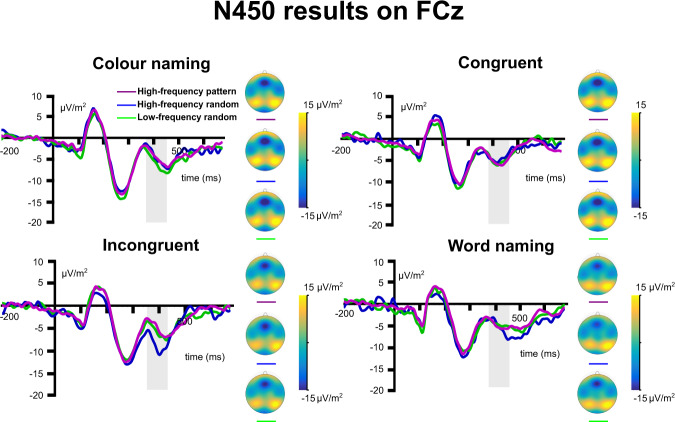
N450 data on channel FCz. Time point zero represents the stimulus presentation. The analysed time window (380–480 ms) is marked with a shaded area. The N450 is organised into four conditions: colour naming, congruent, incongruent, and word naming. The data is presented as a function of triplet types: high-probability pattern (purple), high-probability random (blue), and low-probability random (green). The scalp topography plots show the distribution of the mean activity of each presented condition. The presented result is based on *N* = 31 participants’ data.

Next, the mean amplitude of the N450 in the time window of 380–460 ms was analysed in a two-way repeated-measures ANOVA with predictability and condition as within-subject factors. Grand averages of N450 waveforms on the channel FCz are presented in Fig. 3. The main effects and interactions are summarised in Table 3. The predictability by condition (*F*(6, 180) = 2.47, *ε* = 0.876, *p* = 0.032, *η*_p_^2^ = 0.076) interaction was significant. The interaction effect was further analysed below.

### Statistical and rule-based learning effects on the N450

The main effects and interactions are summarised in Table 2. The *statistical learning* effect on the N450 was larger in incongruent (−2.94 µV/m^2^ ± 1.26) than in colour naming (1.44 µV/m^2^ ± 1.09, *p* = 0.046) condition. The other pair-wise differences (*p*s > 0.281) were not significant. In case of *rule-based learning*, the pair-wise differences were not significant (*p*s > 0.066). That is, the statistical learning effect in the N450 was larger in incongruent than in the colour naming (neutral) condition (cf. RT results).

In sum, ERP analyses showed that conditions were differentiated from each other in different processing stages: switching between the perceptual and semantic response dimensions presented a significant effect on the P3 but not on the N450, while stimulus conflict (condition) was a significant effect on the N450 but not on the P3. Crucially, the N450 was modulated by the interaction between predictability and condition. The follow-up analysis showed larger statistical learning in incongruent trials Thus, the ERP analyses confirmed the behavioural results of interaction between predictability and condition. The interaction effect was significant only in N450 but not in P3, which suggests that the interaction is specific to the type of regularity (statistical learning), conflict condition (incongruent), and processing stage (N450). Next, the onset latency of the N450 in the time window of 380–460 ms was analysed in a two-way repeated-measures ANOVA with predictability and condition as within-subject factors. No significant effects were obtained (Table 3).

## Discussion

We have investigated how incidental learning modulates cognitive control at the levels of behavioural adaptation and related neurophysiological signatures. It was suggested that regularities in the stimulus stream modulate cognitive control through binding between stimulus and control states^8–10,13,16,52^. This is the first study to provide evidence for this account by testing specific stages of cascaded processes (i.e., the components of N450 and P3) coded in the neurophysiological signal. We suggest that it is crucial to consider the type and functionality of the learnt regularities in the interaction between incidental learning and cognitive control. According to follow-up experiments (Supplementary Results), the interaction between learning and control functions may be smaller or absent under certain conditions, which supports the multifactorial nature of this relationship.

Participants performed a Stroop task in which the task-relevant colour information was presented alone (colour naming), in line with the semantic information (congruent), or in conflict between them (incongruent). Response speed for the semantic information alone was also assessed (word naming). Compared to neutral colour naming, responses were slower if perceptual and semantic stimulus dimensions were in conflict (incongruent) or if the response dimension needed to be shifted from colour to semantic information (word naming). In contrast, an alignment between the two dimensions (congruent) facilitated the responses. Slower reactions in incongruent than in congruent trials suggest that a general congruency effect (i.e., the Stroop effect) was observable in the experiment^15,39,55^. Unexpectedly, incongruent trials were not significantly slower than colour naming trials, which could be explained by these conditions’ involvement in the interaction effect (see below).

Unbeknown to the participants, trials were presented according to two kinds of regularities. The first one determined an alternating position in the stimulus sequence (rule-based learning), while the second one predicted the probability of non-adjacent transitions (statistical learning, see also Fig. 1). Importantly, when the demand for cognitive control was the largest, participants’ responses differed between low-probability and high-probability stimulus continuations. This interaction between incidental sequence learning and cognitive conflict was specific to statistical learning and for the first half of the experiment. This specificity might suggest that there is no universal information exchange throughout the task between sequence learning and cognitive control. Namely, statistical learning but not rule-based learning significantly modulated responses in incongruent trials. This difference between the two forms of sequence learning is surprising, considering that statistical learning is a more bottom-up, automatic process compared to rule-based learning^27^. According to our expectations, rule-based learning was a more likely candidate to interact with (top-down) cognitive control. Two aspects of how statistical learning was implemented in the current study might explain the results. It was suggested that the complexity of the probabilistic relations could involve additional processes beyond statistical learning. Specifically, non-adjacent dependencies require the partial inhibition of the intervening item^24^. As statistical learning scores in the current study were based on non-adjacent transitions, an interaction between statistical learning and cognitive control might be plausible. Additionally, statistical learning typically evolves faster than rule-based learning in probabilistic sequences^26,33^. It is possible, that longer exposure to the sequence is needed to induce interaction between rule-based learning and cognitive control. Notably, statistical learning modulated incongruent responses in the first half of the task only. As the response cost of incongruent trials also decreased from the first to the second half of the experiment, it is possible that the functionality to predict incongruent trials also attenuated.

The interplay between control states and regularities has been further analysed at the level of neurophysiology. The P3 and the N450 components were sensitive to different aspects of task demands, which confirms their interpretation as different stages in the chain of processing the control-related information^39,42,56^. The P3 amplitude was smaller in word naming than in congruent or incongruent conditions. Word naming was the only condition in which the predominant response dimension was semantic instead of perceptual. As this condition was also characterised by slower responses and a lower accuracy rate than in the other trials, we suggest that P3 amplitude modulations reflected the increased effort to retrieve the less frequently used S-R associations^48–50^. However, the learning and retrieval of S-C associations did not modulate the P3, unlike the S-R associations in sequential regularities^38,45–50^. In previous sequence learning studies, the P3 amplitude differed between more predictable and less predictable trials^28,38,45–50^. The lack of significant effect of predictability either alone or in interaction with the conflict conditions signals caution on interpreting learning effects in the current study. At the same time, it is feasible that S-C learning does not involve the same ERP modulations as S-R learning. In sum, P3 amplitude changes did not reflect learning effects, however, the component’s amplitude decreased as an indication of the increased difficulty of response selection^42,43,51^.

In contrast, changes in the N450 amplitude followed multiple task dimensions. The sensitivity to the cognitive conflict was confirmed by an increased amplitude in incongruent trials. The incongruent effect on the N450 was larger and conflict detection more pronounced when events could be predicted by statistical learning. Next, we discuss these findings in regard to the interplay between sequence learning and cognitive control.

Some accounts promoted that incidental sequence learning and cognitive control compete for the same neural resources, and therefore, neither function can benefit from the other^53,57–61^. At first blush, both the behavioural and neurophysiological results support competition between learning and conflict processes. Participants showed engagement of cognitive conflict as evidenced by the Stroop effect. However, neither the predictability main effect nor the predictability by period interaction was significant. Traditionally, these effects were taken as markers of learning S-R contingencies in an alternating sequence learning task^21,22^. In the current study, sequential regularities predicted the upcoming control state but not the colour of the stimuli. Therefore, participants could not anticipate the response itself, however, learning could have prepared them for the type of response selection and the associated demand for top-down engagement. That is, predictable trials presented a cognitive advantage in terms of response preparation for all control states. However, this advantage did not manifest as an effect of predictability. Thus, one might conclude that in presence of an intentional (overt) cognitive control task, parallel processing of incidental (covert) sequence learning is not possible. Nonetheless, two aspects should be considered before this conclusion can be reached: (i) the circumstances under direct evidence of competition that were shown in previous studies, and (ii) the type of representations that should be detected in a learning effect. Originally, a competition was suggested between memory systems: automatic processes mediated by the striatum and voluntary, attention-dependent processes mediated by the prefrontal and medial temporal lobes^14,57,60,62^. Later, this competition theory was extended to a resource conflict between cognitive control and procedural sequence learning^53,58,59,61,63^. Notably, sequence learning improved when control-related prefrontal functions were attenuated by hypnosis^59^ or brain stimulation^53,58,63^. In contrast, the current study investigated the interplay between sequence learning and conflict without an attempt to attenuate either learning or control functions. Moreover, competition in previous research was demonstrated by measuring the two functions separately^53,58,59,61,63^. It is conceivable that interaction could only be detected when sequence learning and cognitive control are measured in the same task or situation^7,10^. This difference directly leads us to the second important aspect: what is being learnt in a sequence.

In the current study, the sequence could be used to predict the next control state. Since the importance of an S-C event file is in recognising the need for cognitive control^10^, it is not necessarily expected to be shown as a predictability effect or predictability by period interaction. Rather, the type of predictability, the Stroop condition, and the task period should determine together whether incidental sequence learning can benefit from the control state and vice versa. Thus, the obtained three-way interaction (also in the replication study, see Supplementary Results) support our hypothesis that participants can learn both statistical and rule-based regularities of S-C episodes. However, the P3 component did not show learning effects, as we expected based on previous studies^38,45–50^. This difference might indicate that learning of abstract regularities involves other neurophysiological mechanisms than learning of physical properties, hence, the P3 is not sensitive to both types of memories. Alternatively, since S-C learning is not expected to be expressed without the presence of conflict, and the P3 was not sensitive to stimulus conflict, this component also cannot be sensitive to the different types of regularities. In this scenario, contrary to our original expectations, the conflict-related N450 should be considered sensitive to learning S-C associations on the neurophysiological level. In contrast to the P3, the N450 showed an interaction between sequence learning and cognitive conflict.

Interactions between learning and cognitive conflict occurred both in the behavioural and neurophysiological data. Thus, the current results are in line with the notion that by binding goal representations (C) and contextual information (S) together, S-C associations modulate behaviour^1,9,10,13^. It has been proposed, that S-C associations have a role in control functions only when less demanding processes (such as response priming, S-R predictions, etc.) are not sufficient to direct response selection^8,10^. Therefore, the current study investigated this prediction under challenging conditions. Namely, the Stroop task had four conditions and four related control states, therefore, predicting the correct state might have reduced the cost of adaptation more than in binary-outcome (congruent vs. incongruent) versions of the task. Indeed, when S-C associations were highly predictive (80% probability) but participants only needed to switch between two control states, error rates were minimal^20^. Moreover, half of the trials were incongruent which might have prevented the participants from effectively altering their response preparations. If the possibility of a conflict is always high, there is little benefit in ever letting the guard down. Consequently, S-C learning did not occur^20^. Of note, the current study did not aim to test the design of Jiménez et al.^20^ with four instead of two conflict conditions. Nevertheless, the difference in task difficulty and error rate should be mentioned given its potential significance in cooperative interaction between learning and cognitive control^8,10^. Along these lines, in Experiments 1 and 2, learning and control functions showed interactive effect only when a high level of control was necessary (incongruent condition) and therefore, response selection was challenging. This pattern did not only confirm our hypothesis but is also similar to previous studies showing that the incongruent response cost is smaller in predictable than in unpredictable trials^16^ and sequence learning is enhanced when S-R mapping is incompatible in contrast to compatible configurations^52^. Importantly, this similarity between how S-R and S-C predictions contribute to the congruency effect supports the idea that lower and global level of binding represents compatible representation systems that respond to experimental effects in similar ways^10^.

Importantly, the obtained interaction in the behavioural and neurophysiological data between statistical learning and cognitive control highlights the potential complexity of integrating S-C associations. Previously, predictability was quantified in Stroop tasks as a first-order transition between two consecutive trials^16,20^. The current study investigated the role of non-adjacent, second-order transitional probabilities. Control states were predictable as a condition of the previous two events (high vs low-probability triplets). Triplet probabilities were quantified as moving dependencies throughout the stimuli. That is, a sequence item could always be categorised as the first part of the triplet (predictor item), as a second part (interleaving item), or as a final part (predicted item). Interestingly, learning of non-adjacent transitions was effective in enhancing conflict detection for incongruent trials (i.e., larger N450) and consequently, reducing response cost. That is, the detection and acquisition of second-order transitions seemed to allow the interplay with prefrontal functions, which further promotes the view that this type of dependency calls for inhibitory activity^24^.

The current results fit into a larger picture of statistical information modulating attention, inhibitory control, or linguistic processes. For instance, learning the distributional statistics of conflict levels modulate the allocation of attentional resources during distractor suppression^17,64–67^. Similarly, the distribution of different task conditions can be used to prepare for a potential change in task switching^68^. Mediating between inhibition and sequence learning is also important in processing complex syllable strings^24^ or syntax^69,70^ with non-adjacent interrelations, or other aspects of nested predictabilities in language^29^. In sum, learning probability information contributes to various forms of top-down engagement. The current study provides expands this list by showing an interaction between statistical learning and conflict monitoring at the neurophysiological level.

The follow-up studies outlined in the Supplementary Materials provided important details on the generalisability of the results. While the main finding was replicated in Experiment 2, different control conditions in Experiment 3 or a different sequence in Experiment 4 did not yield significant interaction between predictability and cognitive control. In general, the differences (and similarities) across the experiments might be related to the relatively small interaction effect. Notably, a large effect was not expected given that participants were unaware of the learning task; the sequence followed a complex alternating structure; and the sequence consisted of abstract items (congruency) instead of easily learnable physical features (e.g., colours). Further studies are warranted to dovetail the nature of the interaction between statistical learning and conflict monitoring, including the aspects that might enlarge the interaction.

One such aspect is the length of exposure to the sequential regularities. It is possible that the amount of time needed to detect rule-based learning effects in S-R sequences is not enough when more abstract features need to be integrated. It is worth noting that even when S-R contingencies are to be learnt, better detection of rule-based learning is often boosted by cues on the serial order^22^. However, to keep the covert nature of the sequence, we did not introduce differences between the presentation of pattern and random events. Notably, explicit pre-cues on trial congruency modulate cognitive control in the upcoming trial^71^, which suggests that control processes can occur before the stimulus onset. Nevertheless, longer training and cued sequences in future studies would provide an important contribution to the behavioural dissociation between statistical and rule-based control state predictions.

Another aspect is the potential process-specific relationship between cognitive control and incidental sequence learning. In Experiments 1 and 2, the interaction occurred between cognitive control and statistical learning but not with rule-based learning. In Experiment 3, no interaction was observed, however, significant statistical learning occurred without a rule-based learning effect. Crucially, cognitive control is also non-unitary^6^. The significant interactions in Experiments 1 and 2 occurred in a configuration that included not only the manipulation of stimulus conflict but also switching between the frequent perceptual and the rare semantic response dimension. In contrast, no interaction occurred in Experiment 3, when the fourth condition also required perceptual decision-making. The differences between Experiments 1 and 2 and Experiment 3 may suggest that the nature of stimulus conflict and response inhibition determines if and how statistical learning interacts with cognitive control. However, switching the word naming condition to object colour naming did not only change the conflict configuration of the task, it also decreased the overall complexity. In Experiment 3 participants were the fastest and the most accurate compared to Experiments 1 and 2. As a challenging environment is thought to induce an interaction between sequence learning and cognitive control^8,10^, it is unclear whether Experiment 3 can be interpreted as a process-specific or an effort-related difference to Experiments 1 and 2. Another noteworthy difference occurred between Experiments 1-2 and Experiment 4, which tested the generalisability of the original findings to another sequence and revealed no significant interaction between predictability and condition. Importantly, the difference between Experiments 1-2 and Experiment 4 was not limited to the significance of the interaction but was also present in the main effects. Specifically, the predictability main effect was significant in Experiment 4 unlike in Experiments 1-2. Post-hoc tests showed significant statistical learning but not rule-based learning effect. The condition was also significant. However, the pair-wise comparisons did not show response cost for incongruent trials. Thus, while there was no significant interaction effect in the last experiment, the significant main effects also did not appear to be typical: rule-based learning without statistical learning and a congruent response benefit without an incongruent response cost. Also considering the smaller sample size in Experiment 4, this leaves the question of sequence-specificity inconclusive.

In sum, main finding was that predictability of a sequence influenced cognitive control, but the strength of the effect varied across different experiments. In Experiment 2, the main finding was replicated, suggesting that it was reliable. However, in Experiment 3 and Experiment 4, the interaction between predictability and cognitive control was not significant, indicating that the relationship may be weaker or absent under certain conditions. Differences (and similarities) across the four experiments might suggest that the duration of the task, the complexity and type of the conflict conditions, and tentatively the sequence-specific interrelations can modulate the interaction between incidental sequence learning and cognitive control. The experiments presented in the current study fit into the larger picture of how multifactorial can be a real-life adaptation to an ever-changing environment^10,54^.

We tested possible interrelations between incidental sequence learning and cognitive control. To achieve this, we designed a task in which control demands could be predicted by sequential regularities. Participants’ responses adapted to the conflict between perceptual and semantic information; however, they did not express control-independent learning of the S-C regularities. Statistical learning of S-C associations was enhanced in high stimulus conflict at the levels of response times and the amplitude of the conflict monitoring-related neurophysiological signal. Thus, the nature of conflict, the type of sequence learning, and the stage of information processing determine together whether cognitive conflict and sequence learning support or compete with each other. Specifically, statistical learning has the potential to modulate conflict monitoring. Follow-up experiments demonstrated the limits of the interaction’s generalisability suggested that the relationship between cognitive control and statistical learning may be process-specific and influenced by the nature of stimulus conflict and response inhibition. The experiments fit into a larger framework of how people adapt to an ever-changing environment and advocate that real-life adaptations are multifactorial. We suggest that connecting the fields of cognitive control and incidental learning is essential to achieve a synergistic view of adaptive behaviour.

## Methods

### Participants

*N* = 33 young adults participated in Experiment 1, who were recruited from the voluntary pool for behavioural studies at the TU Dresden (26.6 years ± 6.6, 13 female, 18 male). Since the hypotheses concerned the detection of the learning effect, this parameter was decisive in sample size. Previous reliability analysis showed that stable learning effects can be detected from *N* > 21 in the case of alternating sequence presentation^72^. However, since the paradigm was combined with a Stroop task in a novel way, we aimed to increase this number, and have at least 30 participants for the final analysis. We estimated a 10% loss in an EEG experiment, therefore, 33 participants were recruited. Due to incomplete testing in one case and low data quality in another case (see *EEG recording and analysis*), N = 31 participants’ data were analysed. Experiment 2 was conducted to internally replicate the behavioural results of Experiment 1 (see details in Supplementary Results). *N* = 30 participants were recruited, from which *N* = 28 participants’ data were analysed (due to incomplete testing in two cases). Experiment 3 (Supplementary Results) was conducted with *N* = 20 newly recruited participants. Experiment 4 was conducted with *N* = 20 newly recruited participants to investigate the generalisability of the findings of Experiments 1 and 2 to other sequential regularities. There is no overlap between the groups of Experiments 1–4. All participants in Experiments 1–4 had a normal or corrected-to-normal vision, including colour discrimination. None of the participants reported taking centrally acting medication or having a history of neurological or psychiatric conditions. Participants were either native in German or had a similar level of proficiency (based on self-reports). Written informed consent was provided prior to enrolment, and participation was rewarded with 10€. The study was approved by the local ethical review committee and was conducted in accordance with the Declaration of Helsinki.

### Stimuli, task, and procedure

Participants completed a paradigm that combined an overt (Colour-Word Stroop-Task)^73,74^, and a covert task (Alternating Serial Reaction Time, ASRT)^21,75^. The task is shown in Fig. 1.

Participants saw either colour words in German (ROT: red, BLAU: blue, GELB: yellow, GRÜN: green) or coloured asterisks on the centre of the display. Participants were asked to press a button on the keyboard corresponding to the colour of the word or asterisks, irrespective of the meaning of the word. However, if the word was presented in black, they were asked to press the button that corresponds to the meaning of the word. In 25% of the trials, the word was written in the corresponding colour (i.e., ROT in red, *congruent condition*). In 25% of the trials, the word was written in a mismatching colour (i.e., ROT in blue, *incongruent condition*). In 25% of the trials, the word was presented in black (*word naming condition*), and in the remaining trials only coloured asterisks were presented (*colour naming condition*). These trial types are henceforth referred to as conditions. In each trial, one of the four possible colours was randomly selected.

The covert task followed the structure and timing (including the response window) of a previous version of the ASRT task that had been optimised for EEG recordings^33^. Unbeknown to the participants, stimuli were presented according to an eight-element sequence. Within the sequence, pattern (P) and random (r) items alternated with each other. That is, a sequence of 1-r-2-r-3-r-4-r determined the order of the trial types [1 = congruent, 2 = incongruent, 3 = word naming, 4 = colour naming], interleaved by randomly selected trial types. One of the possible permutations of these of the stimulus sequence was selected for the study and presented to the participants in a pseudo-random manner^21,33,38^. Importantly, the alternating structure used in the current study leads to probability differences between chunks of three successive trials (triplets). In the case of the above-mentioned example sequence, a triplet starting with 2 and ending with 1 is a high-probability triplet that either occurred as a result of a P-r-P or an r-P-r structure. In contrast, a triplet starting with 1 and ending with 2 is a low-probability triplet that could only have an r-P-r structure (see Fig. 2). The distinction between low-probability and high-probability triplets does not only describe the distributional but also the second-order transitional probabilities in the task. That is, a final item of a high-probability triplet is a highly predictable continuation of the first item, while a low-probability triplet’s first item does not carry such anticipatory information. For instance, a triplet starting with 1 has a 62.5% probability of ending with 2, while a triplet starting with 2 has only a 12.5% probability of ending with 1. As a consequence of the continuous stimulus presentation and the unmarked triplet structure, each trial can be categorised as the third item of a high-probability or a low-probability triplet. Furthermore, triplets were organised as moving chunks across the stimuli: the third item of a triplet was also a second item of the next triplet and the first item of the subsequent one^54,75^. The task consisted of 16 high-probability triplets, which individually occur five times more often than the 48 low-probability triplets. Moreover, the combination of triplet probability (high-probability versus low-probability) and position in the sequence presentation (pattern versus random) leads to three trial types of sequence regularities: *high-probability pattern* (50% of all trials), *high-probability random* (12.5% of all trials), and *low-probability random triplets* (37.5% of all trials). The trial types of cognitive conflict were equally distributed among the trial types of sequence regularity, that is, 25% of high-probability triplets were congruent, 25% incongruent, 25% word naming, 25% colour naming, etc.

The timing of the task followed previous EEG studies of the ASRT (Kóbor et al., 2018; 2019)^33,38^. Participants saw either a colour name or a string of coloured asterisks on the centre of the screen for 200 ms. It was followed by a blank screen for 500 ms or until the participant pressed a response button. If the response was incorrect, a blank screen was presented for 500 ms after the response onset, followed by an “X” on the centre of the display for another 500 ms. If the participant did not respond in the trial, the 500 ms-long blank screen was followed by an “!” in the centre of the screen for 500 ms. After a correct response or the incorrect/missed response feedback, a 700-ms-long RSI was introduced.

The task presentation was organised into blocks, each of them containing 85 trials. Each block started with 5 warm-up trials that were excluded from the analyses, and 10 repetitions of the eight-element sequence. After completing a block, participants received feedback about their mean RT and accuracy in the block, which was presented for 4000 ms. Between blocks, participants could take a short break. The task consisted of 20 blocks in total. During the behavioural analysis, the first 10 blocks were collapsed as first half of the task, and the remaining 10 blocks were collapsed as the second half of the task. The experiment lasted about 1–1.5 h, including dimming and removing the EEG cap. Task presentation was written and controlled by Presentation software (Neurobehavioral Systems). Stimuli were displayed on an LCD screen with a viewing distance of 80 cm.

### EEG recording and analysis

The EEG was recorded from 60 Ag/AgCl electrodes mounted in an equidistant way on an elastic cap (EasyCap, Germany). The ground and reference (Fpz) electrodes were placed at coordinates *θ* = 58, *φ* = 78 and *θ* = 90, *φ* = 90, respectively. A BrainAmp amplifier and the Brain Vision Recorder 1.2 software (Brain Products, Germany) were used with a sampling rate of 500 Hz. Offline, data were down-sampled offline to 256 Hz. Impedances were kept below 5 kΩ. The recorded EEG signal was pre-processed using “automagic”^76^ and EEGLAB^77^ in Matlab 2019a (The MathWorks Corp.). First, flat channels were removed and the EEG data were re-referenced to an average reference. Next, the “PREP pipeline”^78^ was applied to remove line noise at 50 Hz by using a multitaper algorithm. PREP also removed contaminations by bad channels and consequently created a robust average reference. Next, EEGLAB’s “clean_rawdata” was applied. This included detrending the EEG data with an IIR high-pass filter of 0.5 Hz (slope 80 dB). Flat-line, noisy, and outlier channels were detected and removed by “clean_rawdata”. Time periods with abnormally strong power (i.e., larger than 15 SD relative to calibration data) were reconstructed by using Artefact Subspace Reconstruction (ASR; burst criterion: 15)^79^. Time windows that could not be reconstructed were removed. A low-pass filter of 40 Hz (sinc FIR filter; order of 86)^80^ was applied. EOG artefacts were removed using a subtraction method^81^. Muscle, heart, and remaining eye artefacts were automatically classified and removed by using an independent component analysis (ICA) with ICLabel^82^. Removed and missing channels were interpolated (average of 0.82 channels) according to the spherical computation. As a final step of pre-processing, the data were inspected visually and epochs with bad data were removed. This step was only necessary if non-repeating noises (e.g., due to movement) remained in the data. Segmentation of the continuous EEG data was performed in two consecutive steps. First, segments were created separately for the first and second half of the task, each consisting of 10 blocks. The length of these acquisition periods corresponds to previous EEG analyses that used the ASRT task and showed reliable learning effects^37,83,84^. Subsequently, segments were averaged for all combinations of the sequence regularity (predictability in the ASRT) and Stroop conditions, namely (average and standard deviation of the numbers of trials reported): high-probability pattern congruent (164 ± 25), high-probability pattern incongruent (123 ± 33), high-probability pattern word naming (138 ± 29), high-probability pattern colour naming (156 ± 27), high-probability random congruent (32 ± 6), high-probability random incongruent (33 ± 9), high-probability random word naming (30 ± 8), high-probability random colour naming (43 ± 7), low-probability random congruent (82 ± 13), low-probability random incongruent (66 ± 17), low-probability random word naming (73 ± 14), low-probability random colour naming (73 ± 13). These combinations were named *event types*. Group-level ERP waveforms were calculated separately for each event type in the acquisition phases. These waveforms were visually inspected to determine if typical ERP correlates of a Stroop task^39^ emerged and to determine the latency range that might vary as a function of event types. This was necessary since the combination of an ASRT and a Stroop task was unprecedented, and therefore, the characteristics of the ERP correlates were unknown. Based on the known characteristics of the components, parietal channels were considered for the P3^38,51^ and frontocentral channels for the N450^39,85,86^. The P3 was quantified as mean amplitude between 280 and 380 ms after stimulus onset on channel P1. The N450 was quantified as mean amplitude between 380–480 ms after stimulus presentation on the channel FCz.

### Statistics and reproducibility

Statistics were performed in JASP version 0.16.2 (JASP Team). We analysed the interaction between learning (predictability) and cognitive control (condition) by performing repeated measures ANOVAs. First, the full factorial design of the experiment was analysed with the within-subject factors of predictability (as triplet types: high-probability pattern, high-probability random, low-probability random), condition (congruent, incongruent, word naming, colour naming), and period (first half and second half). If a significant interaction occurred that included the factors of predictability and condition, follow-up analyses were conducted to quantify how predictability could affect RT, accuracy, or mean amplitude of the N450 and P3 components in different types of conflict situations. These post-hoc analyses were done in accordance with previous studies that used the ASRT^22,26,32,53,54^. Specifically, we analysed whether the difference between high-probability random and low-probability random trials (*statistical learning*) and the difference between the high-probability pattern and high-probability random trials (*rule-based learning*) was dependent on the condition. If the omnibus ANOVA showed a significant interaction between predictability and condition, repeated measures ANOVAs were performed with condition (congruent, incongruent, word naming, colour naming) as a within-subject factor and either statistical learning or rule-based learning score as a dependent variable. Similarly, if the omnibus ANOVA showed a significant interaction between predictability, condition, and period, repeated measures ANOVAs were performed with condition (congruent, incongruent, word naming, colour naming) and period (first half, second half) as a within-subject factor and either statistical learning or rule-based learning score as a dependent variable. Bonferroni-corrected pairwise differences were reported in the Results section. This was done to limit the number of post-hoc tests and still ensure the correction for multiple comparisons. The Huynh-Feldt epsilon was considered as a correction for lack of sphericity in the ANOVA models. Effect sizes are reported as partial eta-squared. Post hoc pairwise comparisons were Bonferroni-corrected.

*Statistical learning* was calculated as the difference between high-probability random and low-probability random trials in RT, accuracy and mean activity. *Rule-based learning* was calculated as a difference between the high-probability pattern and high-probability random trials in RT, accuracy and mean activity. For all analyses, two types of low-probability triplet configurations were excluded, since previous studies^54,87^ showed frequent response bias in these combinations: repetition (e.g., 2r-2P-2r, 3r-3P-3r) and trills (e.g., 1r-2P-1r, 4r-3P-4r). Therefore, repetitions and trills were not part of the low-probability random triplet averages and segments for the behavioural and EEG analyses. Given the characteristics of the N450 component in the different conditions (Fig. 3), latency of the component was analysed in an exploratory fashion. To quantify the onset latency of the N450, the fractional peak method was used^88^. The onset of the component was marked at the time point when 30% of the peak amplitude was reached.

### Reporting summary

Further information on research design is available in the Nature Portfolio Reporting Summary linked to this article.

## Supplementary information

Supplemental Material

Reporting Summary

## Data Availability

De-identified human behavioural data and neurophysiological datasets have been deposited at Open Science Forum https://osf.io/yn4fg/?view_only=b1f8bb4be0c9458396dbb518829908e8^89^. We used standard software packages as described in the “Methods” section. Neurophysiological datasets in different stages of processing (e.g., raw, pre-processed) are available after specification of requested format upon reasonable request by the lead contact.

## Code availability

No custom code was used to generate or process the data described in the manuscript. A history file that includes the EEG analysis steps after pre-processing has been deposited at Open Science Forum https://osf.io/yn4fg/?view_only=b1f8bb4be0c9458396dbb518829908e8^89^. The file can be opened in Brain Vision Analyzer (Brain Products, Germany).
