# Supplementary material for: A neurophysiological perspective on the integration between incidental learning and cognitive control
Source: Commun Biol. 2023 Mar 27;6:329. doi: 10.1038/s42003-023-04692-7 (PMC10042851; doi:10.1038/s42003-023-04692-7)
Supplement: Supplementary file 1 — Supplemental Material [file 42003_2023_4692_MOESM1_ESM.pdf]

Supplementary Material

A neurophysiological perspective on the integration between incidental learning and cognitive control

Adam Takacs, Christian Beste

Supplementary Results

Experiment 2

In Experiment 2, the task remained identical to Experiment 1, the procedure was changed only in regard to not using EEG caps and recordings during the session. Experiment 2 was conducted to investigate the replicability of Experiment 1 on the behavioural level. That is, the main finding in Experiment 1 (Larger statistical learning in the incongruent condition than in colour naming in the first half of the task) was analysed as a planned contrast. In the first period, participants showed larger statistical learning in the incongruent (-9.4 ms ± 7.5) than in the colour naming condition (11.0 ms ± 4.9); t(27)=2.73, p = 0.011, two-tailed. Thus, the main result of Experiment 1 was confirmed. However, for the sake of completeness, we also report the results in the same structure as we did for Experiment 1. Similar to the main study (Experiment 1), we analysed the roles of predictability (as triplet types: high-probability pattern, high-probability random, low-probability random), condition (congruent, incongruent, word naming, colour naming), and period (1st half and 2nd half).

First, RTs were analysed in a three-way repeated-measures ANOVA with predictability, condition, and acquisition period as within-subject factors. The main effects and interactions are summarized in Table S1. The main effects of predictability ($F(2, 54) = 4.34$, $\varepsilon = .801$, $p = .026$, $\eta_p^2 = .138$), condition ($F(3, 81) = 148.29$, $\varepsilon = .626$, $p < .001$, $\eta_p^2 = .846$) and period ($F(1, 27) = 35.16$, $p < .001$, $\eta_p^2 = .566$) were significant. Participants responded slower to the high-probability pattern than to low-probability random trials (503.1 ms ± 6.4 vs 500.4 ms ± 6.0, $p < .047$). Additionally, responses were slower in word naming (541.5 ms ± 5.9) than in incongruent (499.6 ms ± 7.1, $p < .001$), congruent, (478.3 ms ± 6.8 $p < .001$), or colour naming trials (491.6 ms ± 6.7, $p < .001$). RTs were longer in incongruent than in congruent ($p < .001$) and in colour naming trials ($p = .047$). Additionally, participants responded slower in colour naming than in congruent trials ($p < .001$). Participants became faster for the second period (494.2 ms ± 6.2) compared to the first one (511.4 ms ± 6.8, $p < .001$). Importantly, the three-way interaction between predictability, condition and period was significant ($F(6, 162) = 2.70$, $\varepsilon = .532$, $p = .047$, $\eta_p^2 = .091$). This interaction effect was further analysed in post-hoc analyses.

The main effects and interactions of statistical and rule-based learning analyses are summarized in Table S2. In case of *statistical learning*, the conflict by period ANOVA showed the conflict by period interaction was significant ($F(3, 81) = 3.13$, $\varepsilon = .695$, $p = .049$, $\eta_p^2 = .104$). After Bonferroni-correction, none of the conditions showed modulations between the acquisition periods (*p*s >.074). However, in the second period, statistical learning was larger in congruent (-5.3 ms ± 4.5) than in word naming condition (9.7 ms ± 2.7, $p = .046$). There were no other significant pair-wise differences between conditions in the two periods (*p*s >.066). In case of *rule-based learning*, the conflict by period ANOVA showed that the interaction was significant ($F(3, 81) = 2.98$, $p = .050$, $\eta_p^2 = .099$). In the second period, participants showed larger rule-based learning in the incongruent (-8.4 ms ± 4.6) than in the congruent (9.3 ms ± 3.6, $p = .027$), and larger learning in the word naming (-7.1 ms ± 2.6) than in the congruent condition ($p =.001$). None of the other pair-wise differences was significant (*p*s > .465).

Next, the triplet type by period ANOVAs were conducted separately for the congruent, incongruent and word naming effects. The main effects and interactions are summarized in Table S3. In case of the *incongruency* effect, the main effect of predictability was significant ($F(2, 54) = 3.42$, $p = .040$, $\eta_p^2 = .112$). The incongruency effect was larger in low-probability random trials (13.3 ms ± 3.6) than in high-probability pattern trials (5.4 ms ± 2.7, $p = .023$). The other pair-wise comparisons were not significant ($p > .170$). The triplet type by period

interaction was significant ($F(2, 54) = 3.84$, , $\varepsilon = .865$, $p = .034$, $\eta_p^2 = .124$). In the first period, the incongruency effect was larger in low-probability random (19.3 ms ± 5.5) trials than in high-probability pattern trials (5.5 ms ± 4.0, $p = .031$) and in high-probability random trials (-1.2 ms ± 6.2, $p = .033$). The two high-probability conditions did not differ significantly from each other in the first period ($p = .818$). The pair-wise contrasts in the second period did not reach significance (*p*s >.393).

Accuracy rates were analysed in a three-way repeated-measures ANOVA with predictability, condition, and acquisition period as within-subject factors. The main effects and interactions are summarized in Table S1. The main effects of condition ($F(3, 81) = 33.65$, $\varepsilon = .533$, $p < .001$, $\eta_p^2 = .555$) and period ($F(1, 27) = 97.90$, $p < .001$, $\eta_p^2 = .784$) were significant. Participants made more errors in incongruent (66 % ± 0.03) than in colour naming (76 % ± 0.02, $p < .001$) or in congruent trials (81 % ± 0.03, $p < .001$). Additionally, participants were less accurate in word naming (67.5 % ± 0.3) than in colour naming ($p < .001$) or congruent trials ($p < .001$). Accuracy was lower in colour naming than in congruent trials ($p < .001$). The accuracy did not differ significantly between incongruent and word naming conditions ($p = .999$). Participants became more accurate for the second period (77 % ± 0.02) compared to the first one (68 % ± 0.02, $p < .001$).

The interaction of condition by period was significant ($F(3, 81) = 4.36$, $\varepsilon = .731$, $p = .015$, $\eta_p^2 = .139$). In the first period, participants made more errors in incongruent (61 % ± 0.03) than in colour naming (72 % ± 0.02, $p = .002$) or in congruent trials (78 % ± 0.03, $p < .001$). Additionally, participants were less accurate in word naming (61 % ± 0.03) than in colour naming ($p < .001$) or congruent trials ($p < .001$). Accuracy was lower in colour naming than in congruent trials ($p < .001$). The accuracy did not differ between incongruent and word naming conditions ($p = .999$). Similarly, in the second period, participants made more errors in incongruent (70 % ± 0.03) than in colour naming (79 % ± 0.02, $p < .001$) or in congruent trials (85 % ± 0.03, $p < .001$). Additionally, participants were less accurate in word naming (78 % ± 0.1) than in colour naming ($p < .001$) or congruent trials ($p < .001$). Accuracy was lower in colour naming than in congruent trials ($p < .001$). The accuracy did not differ between incongruent and word naming conditions ($p = .699$). The three-way interaction between predictability, condition and period ($F(6, 162) = 2.49$, $\varepsilon = .883$, $p = .031$, $\eta_p^2 = .084$) was significant. This interaction effect was further analysed in post-hoc analyses. The main effects and interactions of statistical and rule-based learning analyses are summarized in Table S2. In case of *statistical learning*, the condition by period ANOVA showed that the interaction effect was significant ($F(3, 81) = 3.45$, $p = .020$, $\eta_p^2 = .113$). In the first period, participants showed more learning in incongruent (-0.07 % ± 0.2) than in colour naming (0.04 % ± 0.03, $p = .029$) and more learning in incongruent than in congruent trials (0.18 % ± 0.02, $p = .029$). None of the other pair-wise comparisons were significant (*p*s > .140). In *rule-based learning*, the condition by period ANOVA did not show any significant effect.

Next, the triplet type by period ANOVAs were conducted separately for the congruency, incongruency and word naming effects (Table S3). In the incongruency analysis, the main effect of predictability was significant ($F(2, 54) = 5.78$, $p = .005$, $\eta_p^2 = .176$). The incongruency effect was larger in low-probability random trials (-0.13 % ± 0.03) than in high-probability pattern trials (-0.08 % ± 0.02, $p = .003$). The other pair-wise comparisons were not significant (*p*s > .072). The interaction was also significant ($F(2, 54) = 4.30$, $\varepsilon = .769$, $p = .029$, $\eta_p^2 = .137$).

In the first half of the task, the incongruency effect was larger in low-probability random trials (-0.17 % ± 0.03) than in high-probability pattern trials (-0.09 % ± 0.03, $p = .002$) and in high-probability random trials (-0.06 % ± 0.04, $p = .015$). In the second half, none of the comparisons were significant (*p*s > .359). Additionally, the incongruency effect decreased from the first half to the second (-0.09 % ± 0.03, $p < .001$), while the other predictability conditions did not change significantly (*p*s > .283). Finally, in the word naming analysis, the main effect of period was significant ($F(1, 27) = 14.66$, $p < .001$, $\eta_p^2 = .352$): the difference between word naming and

colour naming decreased from the first half of the task to the second one (-0.11 % ± 0.02 vs. -0.05 % ± 0.01).

Experiment 3

In Experiment 3, the task from Experiment 1 was changed in one aspect: instead of word naming, object colour naming was used as the fourth condition. In this condition, participants saw object names on the screen in different colours. They were instructed to press the response button that corresponds to the colour of the letters, irrespective of the meaning of the words. All the words were one-syllable length clothing items that did not have common associations with the four colours: hat (Hut), scarf (Schaal), shirt (Hemd), and skirt (Rock). Thus, in Experiment 4, participants did not have to switch between the dimensions of colour and semantic content when selecting their responses. Instead, the object naming condition was intended to function as a second neutral condition, in which the perceptual and semantic information was not conflicted. In every other detail, including trial number, timing, and procedure, Experiment 3 was identical to Experiments 1 and 2.

Experiment 3 was conducted to investigate the generalizability of Experiment 1 to a Stroop task without the need to switch between response dimensions. We analysed the roles of predictability (as triplet types: high-probability pattern, high-probability random, low-probability random), condition (congruent, incongruent, object colour naming, colour naming), and period (1st half and 2nd half). The obtained results are interpreted in the Supplementary Discussion.

First, RTs were analysed in a three-way repeated-measures ANOVA with predictability, condition, and period as within-subject factors. The main effects and interactions are summarized in Table 1. The main effects of condition ($F(3, 57) = 21.92$, $p < .001$, $\eta_p^2 = .536$) and period ($F(1, 19) = 16.55$, $p < .001$, $\eta_p^2 = .466$) were significant. Participants responded slower in colour naming (479.9 ms ± 7.0) than in congruent (465.6 ms ± 7.1 $p < .001$) or object colour naming trials (472.6 ms ± 7.3, $p = .007$). Participants were slower to respond to incongruent (476.8 ms ± 6.7) than to congruent trials ($p < .001$). Other pair-wise differences between the conditions were not significant (*p*s > .355). Participants became faster for the second period (468.7 ms ± 7.3) compared to the first one (478.8 ms ± 7.3, $p < .001$).

Accuracy rates were analysed in a three-way repeated-measures ANOVA with predictability, condition, and period as within-subject factors. The main effects and interactions are summarized in Table 1. The main effects of condition ($F(3, 57) = 11.69$, $p < .001$, $\eta_p^2 = .381$) and period ($F(1, 19) = 5.46$, $p = .031$, $\eta_p^2 = .223$) were significant. Participants made more errors in colour naming (85 % ± 0.02) than in congruent (88 % ± 0.01, $p = .007$) and in object colour naming trials (90 % ± 0.01, $p = .001$). Additionally, participants were less accurate in incongruent (85 % ± 0.02) than in object colour naming ($p = .002$) and congruent trials ($p < .001$). Other pair-wise differences between the conditions were not significant (*p*s > .107). Responses were more accurate in the second period (88 % ± 0.01) than in the first one (86 % ± 0.02, $p < .031$).

Experiment 4

Experiment 4 was conducted to investigate the generalizability of Experiment 1 to other sequential regularities. First, RTs were analysed in a three-way repeated-measures ANOVA with predictability, condition, and period as within-subject factors. The main effects and interactions are summarized in Table 1. The main effects of predictability ($F(2, 38) = 8.41$, $p < .001$, $\eta_p^2 = .307$), condition ($F(3, 57) = 97.71$, $\varepsilon = .826$, $p < .001$, $\eta_p^2 = .837$) and period ($F(1, 19) = 12.28$, $p = .002$, $\eta_p^2 = .392$) were significant. Participants responded slower to high-probability pattern (508.8 ms ± 6.0) than to low-probability random (505.9 ms ± 6.1, $p = .047$) and to high-probability random (503.1 ms ± 5.9, $p = .005$) trials, while the two random trial type did not differ significantly from each other ($p = .222$). Thus, the effect indicated a

significant rule-based learning in the absence of a significant statistical learning effect. Additionally, responses were slower in word naming (543.4 ms ± 5.5) than in incongruent (497.6 ms ± 6.9, $p < .001$), congruent (487.1 ms ± 6.5 $p < .001$), or colour naming trials (495.5 ms ± 6.5, $p < .001$). Participants were slower to respond to colour naming than to congruent trials ($p = .021$). Other pair-wise differences between the conditions were not significant (*p*s > .060). Thus, congruency and word naming effects were significant in Experiment 3, however, there was no significant incongruency effect. Finally, participants became faster for the second period (502.2 ms ± 6.3) compared to the first one (509.7 ms ± 5.7, $p = .002$). Accuracy rates were analysed in a three-way repeated-measures ANOVA with predictability, condition, and period as within-subject factors. The main effects and interactions are summarized in Table 1. The main effects of condition ($F(3, 57) = 22.15$, $\varepsilon = .677$, $p < .001$, $\eta_p^2 = .538$) and period ($F(1, 19) = 38.02$, $p < .001$, $\eta_p^2 = .667$) were significant. Participants made more errors in incongruent (70 % ± 0.03) than in colour naming (80 % ± 0.02, $p = .004$) and in congruent trials (85 % ± 0.02, $p < .001$). Additionally, participants were less accurate in word naming (71 % ± 0.04) than in colour naming ($p = .001$) and congruent trials ($p < .001$). Accuracy was lower in colour naming than in congruent trials ($p < .001$). Participants' accuracy did not differ significantly between incongruent and word naming conditions ($p = .999$). Responses were more accurate in the second period (81 % ± 0.02) than in the first one (72 % ± 0.02, $p < .001$).

Supplementary Tables

Supplementary Table 1. Results of three-way repeated-measures ANOVAs with predictability, condition, and period as within-subject factors for RT and accuracy data, separately for Experiments 2, 3 and 4. Significant main effects and interactions are boldfaced and detailed in the Supplementary Results.

		EXPERIMENT #2				EXPERIMENT #3				EXPERIMENT #4			
	**Factor**	***F***	**ε**	***p***	**ηp2**	***F***	**ε**	***p***	**ηp2**	***F***	**ε**	***p***	**ηp2**
**Behaviour RT**	**Predictability**	4.34	0.801	**0.026**	0.138	1.03	-	0.368	0.051	8.41	-	**< .001**	0.307
	**Condition**	148.29	0.626	**< .001**	0.846	21.92	-	**< .001**	0.536	97.71	0.826	**< .001**	0.837
	**Period**	35.16	-	**< .001**	0.566	16.55	-	**< .001**	0.466	12.28	-	**0.002**	0.392
	**Predictability X Period**	0.11	0.752	0.843	0.004	0.02	-	0.979	0.001	2.18	-	0.128	0.103
	**Condition X Period**	0.18	-	0.908	0.007	0.88	-	0.455	0.044	0.11	-	0.957	0.006
	**Predictability X Condition**	2.07	0.746	0.081	0.071	0.58	-	0.746	0.030	0.62	-	0.714	0.032
	**Predictability X Condition X Period**	2.70	0.532	**0.047**	0.091	0.91	-	0.492	0.046	0.61	-	0.721	0.031
**Behaviour Accuracy**	**Predictability**	0.05	0.737	0.905	0.002	0.13	0.774	0.875	0.007	1.07	-	0.354	0.053
	**Condition**	33.65	0.533	**< .001**	0.555	11.69	-	**< .001**	0.381	22.15	0.677	**< .001**	0.538
	**Period**	97.90	-	**< .001**	0.784	5.46	-	**0.031**	0.223	38.02	-	**< .001**	0.667
	**Predictability X Period**	0.39	0.867	0.652	0.014	0.16	0.797	0.855	0.008	0.84	-	0.441	0.042
	**Condition X Period**	4.36	0.731	**0.015**	0.139	1.54	-	0.215	0.075	2.37	0.590	0.115	0.111
	**Predictability X Condition**	2.10	0.845	0.068	0.072	0.99	-	0.437	0.049	2.16	0.797	0.068	0.102
	**Predictability X Condition X Period**	2.49	0.883	**0.031**	0.084	1.70	-	0.126	0.082	1.45	-	0.202	0.071

Supplementary Table 2. Results of the condition by period follow-up ANOVAs for statistical learning and rule-based learning for the RT and accuracy in Experiment 2. Significant main effects and interactions are boldfaced and detailed in the Supplementary Results.

		Factor	*F*	*p*	ηp2
Statistical learning	Behaviour RT	**Condition**	2.19	0.095	0.005
		**Period**	0.13	0.726	0.075
		**Condition X Period**	3.13	**0.049**	0.104
	Behaviour Accuracy	**Condition**	1.88	0.140	0.065
		**Period**	0.59	0.450	0.021
		**Condition X Period**	3.45	**0.020**	0.113
Rule-based learning	Behaviour RT	**Condition**	1.63	0.196	0.057
		**Period**	0.01	0.941	0.000
		**Condition X Period**	2.98	**0.050**	0.099
	Behaviour Accuracy	**Condition**	0.59	0.624	0.021
		**Period**	0.17	0.680	0.006
		**Condition X Period**	1.80	0.153	0.063

Supplementary Table 3. Results of predictability by period follow-up ANOVAs for statistical learning and rule-based learning for the RT and accuracy in Experiment 2. Significant main effects and interactions are boldfaced and detailed in the Supplementary Results.

		Factor	*F*	ε	*p*	ηp2
Congruency	Behaviour RT	**Predictability**	2.02	0.812	0.153	0.069
		**Period**	0.30	-	0.589	0.011
		**Predictability X Period**	1.06	-	0.353	0.038
	Behaviour Accuracy	**Predictability**	0.77	0.831	0.446	0.028
		**Period**	0.02	-	0.882	0.001
		**Predictability X Period**	0.30	-	0.744	0.011
Incongruency	Behaviour RT	**Predictability**	3.42	-	**0.040**	0.112
		**Period**	0.01	-	0.931	0.000
		**Predictability X Period**	3.84	0.865	**0.034**	0.124
	Behaviour Accuracy	**Predictability**	5.78	-	**0.005**	0.176
		**Period**	1.30	-	0.263	0.046
		**Predictability X Period**	4.30	0.769	**0.029**	0.137
Word naming	Behaviour RT	**Predictability**	0.62	-	0.540	0.023
		**Period**	0.33	-	0.570	0.012
		**Predictability X Period**	0.56	-	0.573	0.020
	Behaviour Accuracy	**Predictability**	0.16	0.796	0.854	0.006
		**Period**	14.66	-	**< .001**	0.352
		**Predictability X Period**	0.81	-	0.444	0.029

Supplementary Figure 1: P3 data on channel P1.

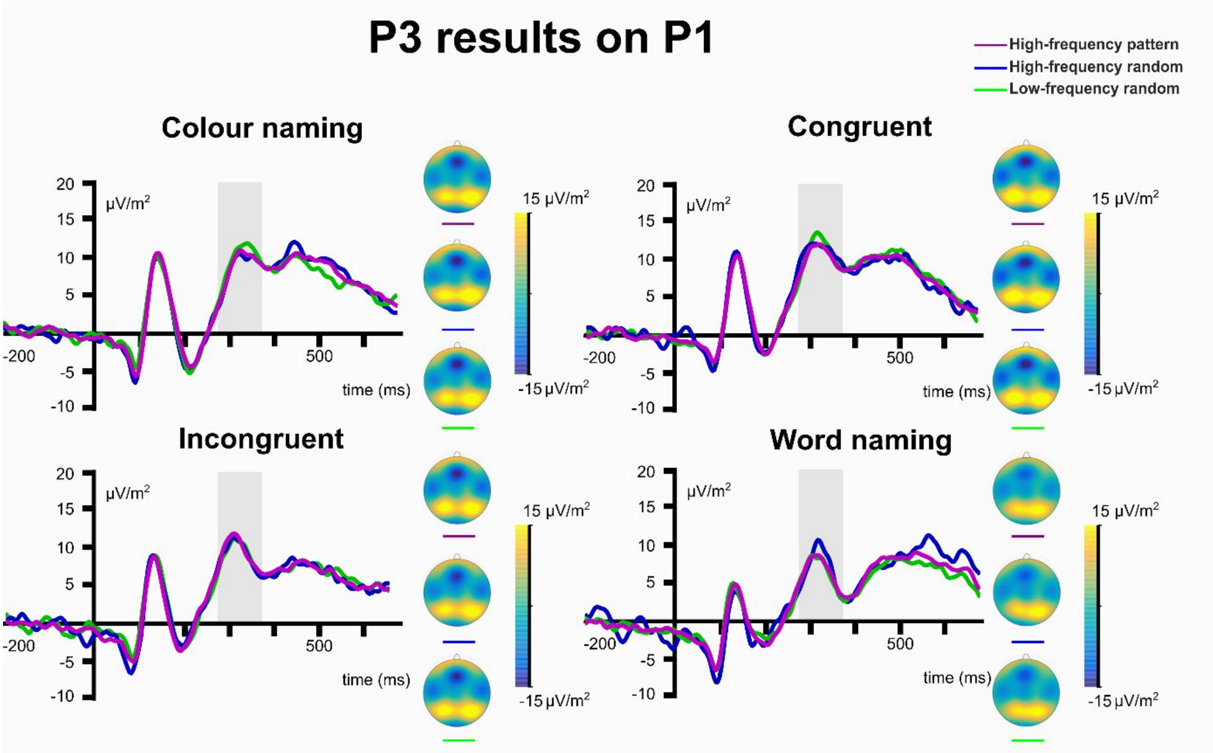

Time point zero represents the stimulus presentation. The analysed time window (280-380 ms) is marked with a shaded area. The P3 is organised into four conditions: colour naming, congruent, incongruent, and word naming. The data is presented as a function of triplet types: high-frequency pattern (purple), high-frequency random (blue), and low-frequency random (green). The scalp topography plots show the distribution of the mean activity of each presented condition.
