# Supplementary material for: A neurophysiological perspective on the integration between incidental learning and cognitive control
Source: Commun Biol. 2023 Mar 27;6:329. doi: 10.1038/s42003-023-04692-7 (PMC10042851; doi:10.1038/s42003-023-04692-7)
Supplement: Supplementary file 2 — Reporting Summary [file 42003_2023_4692_MOESM2_ESM.pdf]

nature portfolio

Corresponding author(s): Adam Takacs

Last updated by author(s): Aug 26, 2022

Reporting Summary

Nature Portfolio wishes to improve the reproducibility of the work that we publish. This form provides structure for consistency and transparency in reporting. For further information on Nature Portfolio policies, see our Editorial Policies and the Editorial Policy Checklist.

Statistics

For all statistical analyses, confirm that the following items are present in the figure legend, table legend, main text, or Methods section.

n/a	Confirmed	
☐	☒	The exact sample size (*n*) for each experimental group/condition, given as a discrete number and unit of measurement
☐	☒	A statement on whether measurements were taken from distinct samples or whether the same sample was measured repeatedly
☐	☒	The statistical test(s) used AND whether they are one- or two-sided<br>*Only common tests should be described solely by name; describe more complex techniques in the Methods section.*
☒	☐	A description of all covariates tested
☐	☒	A description of any assumptions or corrections, such as tests of normality and adjustment for multiple comparisons
☐	☒	A full description of the statistical parameters including central tendency (e.g. means) or other basic estimates (e.g. regression coefficient) AND variation (e.g. standard deviation) or associated estimates of uncertainty (e.g. confidence intervals)
☐	☒	For null hypothesis testing, the test statistic (e.g. *F*, *t*, *r*) with confidence intervals, effect sizes, degrees of freedom and *P* value noted<br>*Give P values as exact values whenever suitable.*
☒	☐	For Bayesian analysis, information on the choice of priors and Markov chain Monte Carlo settings
☒	☐	For hierarchical and complex designs, identification of the appropriate level for tests and full reporting of outcomes
☐	☒	Estimates of effect sizes (e.g. Cohen's *d*, Pearson's *r*), indicating how they were calculated

Our web collection on statistics for biologists contains articles on many of the points above.

Software and code

Policy information about availability of computer code

Data collection	Brain Vision Recorder
Data analysis	Brain Vision Analyzer, Matlab

For manuscripts utilizing custom algorithms or software that are central to the research but not yet described in published literature, software must be made available to editors and reviewers. We strongly encourage code deposition in a community repository (e.g. GitHub). See the Nature Portfolio guidelines for submitting code & software for further information.

Data

Policy information about availability of data

All manuscripts must include a data availability statement. This statement should provide the following information, where applicable:

- Accession codes, unique identifiers, or web links for publicly available datasets
- A description of any restrictions on data availability
- For clinical datasets or third party data, please ensure that the statement adheres to our policy

De-identified human behavioural data and neurophysiological datasets for classification have been deposited at Open Science Forum https://osf.io/yn4fg/?view_only=b1f8bb4be0c9458396dbb518829908e8. We used standard software packages as described in the methods section. Neurophysiological datasets in different stages of processing (e.g., raw, pre-processed) are available after specification of requested format upon reasonable request by the lead contact.

Human research participants

Policy information about studies involving human research participants and Sex and Gender in Research.

Reporting on sex and gender	N = 33 young adults participated in Experiment 1 (13 female, 18 male).
Population characteristics	Participants were neurotypical young adults with the mean age of 26.6 years ± 6.6. All participants in Experiments 1-3 had a normal or corrected-to-normal vision, including colour discrimination. None of the participants reported taking centrally acting medication or having a history of neurological or psychiatric conditions.
Recruitment	Participants were recruited from the voluntary pool for behavioural studies at the TU Dresden. Written informed consent was provided prior to enrolment, and participation was rewarded with 10€.
Ethics oversight	The study was approved by the local ethical review committee (TU Dresden) and was conducted in accordance with the Declaration of Helsinki.

Note that full information on the approval of the study protocol must also be provided in the manuscript.

Field-specific reporting

Please select the one below that is the best fit for your research. If you are not sure, read the appropriate sections before making your selection.

☒ Life sciences ☐ Behavioural & social sciences ☐ Ecological, evolutionary & environmental sciences

For a reference copy of the document with all sections, see nature.com/documents/nr-reporting-summary-flat.pdf

Life sciences study design

All studies must disclose on these points even when the disclosure is negative.

Sample size	N = 33 young adults participated in Experiment 1 (13 female, 18 male). Since the hypotheses concerned the detection of the learning effect, this parameter was decisive in sample size. Previous reliability analysis showed that stable learning effects can be detected from N > 21 in the case of alternating sequence presentation 67. However, since the paradigm was combined with a Stroop task in a novel way, we aimed to increase this number, and have at least 30 participants for the final analysis. We estimated a 10% loss in an EEG experiment, therefore, 33 participants were recruited. Due to incomplete testing in one case and low data quality in another case (see EEG recording and analysis), N=31 participants' data were analysed. Experiment 2 was conducted to internally replicate the behavioural results of Experiment 1 (see details in Supplementary Materials). N = 30 participants were recruited, from which N = 28 participants' data were analysed (due to incomplete testing in two cases). Experiment 3 (Supplementary Materials) was conducted with N = 20 newly recruited participants. There is no overlap between the groups of Experiments 1-3.
Data exclusions	Due to incomplete testing in one case and low data quality in another case (see EEG recording and analysis), N=31 participants' data were analysed.
Replication	Experiment 2 was conducted to internally replicate the behavioural results of Experiment 1 (see details in Supplementary Materials). The behavioural effect of Experiment 1 was successfully replicated.
Randomization	There were no groups, the experiment was conducted as a within-subject design.
Blinding	Investigators were not blinded.

Reporting for specific materials, systems and methods

We require information from authors about some types of materials, experimental systems and methods used in many studies. Here, indicate whether each material, system or method listed is relevant to your study. If you are not sure if a list item applies to your research, read the appropriate section before selecting a response.

Materials & experimental systems

n/a	Involved in the study
☒	☐ Antibodies
☒	☐ Eukaryotic cell lines
☒	☐ Palaeontology and archaeology
☒	☐ Animals and other organisms
☒	☐ Clinical data
☒	☐ Dual use research of concern

Methods

n/a	Involved in the study
☒	☐ ChIP-seq
☒	☐ Flow cytometry
☒	☐ MRI-based neuroimaging
